# Modulation of sirtuin expression by a high-sugar diet and regular swimming trained precedes the loss of kidney function

**DOI:** 10.1590/1414-431X2024e13043

**Published:** 2025-01-31

**Authors:** D.C. Oliveira, D.T. de Oliveira, V.G.O. Neves, I.C. Fernandes, T.A.P. dos Santos, C.M. Carneiro, N.C. Nogueira-Paiva, N.R. Barboza, R. Guerra-Sá

**Affiliations:** 1 Núcleo de Pesquisas em Ciências Biológicas, Programa de Pós-Graduação em Biotecnologia, Universidade Federal de Ouro Preto, Ouro Preto, MG, Brasil; 2 Programa de Pós-Graduação em Ciências Farmacêuticas, Escola de Farmácia, Universidade Federal de Ouro Preto, Ouro Preto, MG, Brasil; 3 Núcleo de Pesquisas em Ciências Biológicas, Programa de Pós-graduação em Ciências Biológicas, Universidade Federal de Ouro Preto, Ouro Preto, MG, Brasil

**Keywords:** Sirtuins, Kidney disease, Obesity, High-sugar diet, Physical training

## Abstract

Sirtuins (SIRTs) are key regulators of cellular metabolism, involved in a wide range of physiological and pathological processes. However, there is scarce knowledge about the effect of sugar consumption and physical activity on SIRTs in kidney disorders. Here, we evaluated the impact of prolonged consumption of an isocaloric high-sugar diet (HSD) and physical training on the modulation of renal Sirts and the link between these alterations and possible obesity-associated kidney damage. Newly weaned male Wistar rats were fed a standard chow diet (STD) or HSD *ad libitum* and then subjected or not to regular workload swimming training for 18 weeks. Morphometric and biochemical parameters were analyzed, and the kidneys were removed for lipid quantification, histological analysis, and for *Sirts1-7* expression. HSD led to the development of obesity, increased serum triglyceride levels, and glucose intolerance, regardless of higher caloric consumption. However, training was able to partially inhibit the HSD-induced obesogenic effect. No changes were identified in kidney mass, lipid content, histology, and creatinine clearance among the groups; these results were associated with a decrease in the renal expression of *Sirt2*-*3* and *Sirt7*; however, training was able to reverse this modulation. The interaction between HSD and training led to an increase in *Sirt4-7*. However, *Sirt1* remained constant among experimental groups. In conclusion, our results indicated that the transcriptional modulation of *Sirts* precedes HSD-induced damage and loss of kidney function, as well as a possible protective adaptive response of physical exercise on long-term *Sirts* expression.

## Introduction

It is widely established that obesity leads to adverse metabolic effects, being an important risk factor for the development of multiple comorbidities, including chronic kidney disease (CKD) (1). Although the pathophysiology of kidney damage associated with excess body adiposity is well characterized and involves a combination of hemodynamic, metabolic, and lipotoxicity changes, the mechanisms of changes at the molecular level underlying this association are still scarce (2). Notably, the sirtuins (SIRTs), a group of deacetylase-dependent proteins of nicotinamide dinucleotide (NAD^+^) and adenine involved in the regulation of metabolism and stress responses (3), have attracted increasing attention of the scientific community due to their regulation of a wide range of physiological and pathological processes in the kidney (4).

In mammals, SIRTs have seven isoforms (SIRT1-7), which are located in different subcellular areas (5,6). SIRT1, SIRT6, and SIRT7 are predominantly present in the cell nucleus, SIRT2 in the cytoplasm, and SIRT3, SIRT4, and SIRT5 in the mitochondria (5,6). In kidney tissue, Sirts are expressed in several compartments playing specific roles. In the glomerulus, SIRT1, SIRT3, and SIRT6 are crucial for podocyte integrity. In nearby glomerular endothelial cells, Sirt1 regulates blood pressure by controlling endothelial nitric oxide (eNOS) synthesis. Sirt1 is also extensively expressed in tubular cells in all segments of the nephron and participates in the control of sodium balance and water reabsorption. SIRT3 is highly expressed in proximal tubule cells, where it is involved in preserving mitochondrial functional integrity. SIRT7 is expressed in the collecting duct, where it controls the acid-base and renal electrolyte management (7,8).

Increasing evidence suggests that dysregulation of renal SIRTs is implicated in the development of renal injuries associated with metabolic diseases. More specifically, renal SIRTs have been shown to have an essential role in the regulation of important cellular processes (including apoptosis, autophagy, fibrosis, inflammation, and oxidative stress) involved in the pathophysiology of kidney damage from metabolic disorders (7,8). However, the understanding of the different functions of SIRTs and the possible modulating role of diet and physical exercise at the renal level are still incipient and need further studies in order to elucidate their effect on the development of renal disorders (8).

Obesity is often caused by hyperactivation of the renin-angiotensin-aldosterone system (RAAS), which can be caused by several factors, including insulin resistance, increased sympathetic activity, and excess adipose tissue among others. This hyperactivation of the RAAS can lead to a series of physiological consequences, including glomerular hyperfunction, vasodilation, and renal damage, through a mechanism that involves increased production of nitric oxide due to increased angiotensin II dysfunction in adipose tissue (9). Of interest, pioneering studies suggest that there is cross-talk between RAAS and SIRTs, and it has been demonstrated that the blockade or activation of SIRTs modulates the expression of RAAS components and *vice versa*. This interaction mutually influences their functions and plays an important role in protecting against cellular dysfunction and damage (through the attenuation of oxidative stress and vascular inflammation), which is possibly an important signaling in kidney injury (10-12).

We have previously shown that prolonged consumption of an isocaloric high-sugar diet (HSD) is capable of inducing obesity and metabolic changes in rats (13). Furthermore, previous results from our research group revealed that the consumption of HSD modulated the expression of a network of genes involved in adipogenesis and also the gut microbiota, but physical activity reverted this diet-induced obesogenic response (14,15). Continuing this line of research and considering that little is known about the effect of high sugar consumption under isoenergetic conditions and physical activity on renal homeostasis and the modulation of *Sirts* by these variables, in the present study, we evaluated the impact of long-term consumption of HSD and regular physical exercise on the development of possible kidney damage/loss of kidney function and the role of *Sirt1-7* expression in this event.

## Material and Methods

### Experimental design

All experimental procedures were performed by following the rules of the Brazilian Guidelines for Animal Experimentation of the National Council for the Control of Animal Experimentation (CONCEA) and approved by the Ethics in the Use of Animals Committee of Federal University of Ouro Preto (UFOP) under protocol No. 2016/26.

Forty newly weaned male Wistar rats (21 days old) were obtained from the Animal Science Center (CCA) of UFOP. The animals were housed in collective cages, under controlled conditions (light-dark cycles of 12/12 h and room temperature of 24±2°C), with free access to water and food.

The animals were randomly divided into four groups: 1) sedentary standard chow diet (S-STD, n=10); 2) trained standard chow diet (T-STD, n=10); 3) sedentary high-sugar diet (S-HSD, n=10); 4) trained high-sugar diet (T-HSD, n=10). The S-STD and T-STD groups were fed a standard chow diet (STD; Nuvilab CR1^®^, Brazil) composed of 57.16% carbohydrate (being 0% added sugar), totaling 308.51 kcal/100 g. The S-HSD and T-HSD groups were fed a high-sugar diet composed of 66.86% carbohydrate (being 36.32% added sugar), totaling 286.36 kcal/100 g. The complete nutritional composition of each diet has been previously reported (13). The animals were fed their respective diets for an experimental period of 18 weeks.

T-STD and T-HSD groups underwent regular swimming training in a collective pool (150×100×60-cm wide) under controlled water temperature (31±2°C) for five days a week. In the first week of the experiment, the animals were adapted to the aquatic environment, as described by Neves et al. (15). The following week, the animals underwent regular swimming training with loads attached to their tail. The training started with a load equivalent to 0.5% of the animal's body weight and increased weekly until reaching 2%. Swimming sessions lasted 60 min a day and were performed five days a week for a period of 18 consecutive weeks.

### Food intake and body weight gain

During the dietary intervention period, the animals were weighed once a week. Body weight gain was calculated as the difference between final body weight and the initial body weight. Dietary intake also was analyzed weekly and total caloric intake was calculated based on dietary intake and the amount of kilocalories (kcal) provided by each diet (STD: 3.08 kcal/g and HSD: 2.86/g).

### Metabolic cages

In the 18th week of the protocol, the animals were housed individually in metabolic cages for 24 h. Food consumption and water intake were also monitored. Urine samples collected over a 24-h period were used to determine urine output and creatinine and protein excretion. The analyses were conducted using a commercial kit from Labtest (Brazil) according to the manufacturer's protocol using an automated analyzer (Wiener Lab, CM 200, Brazil) at the Laboratory of Clinical Analysis of the Pharmacy School of UFOP. The glomerular filtration rate was estimated by creatinine clearance (CrCl) based on serum creatinine levels and 24-h urine flow, reported in mL/min corrected by body mass index (per 100 g of body weight).

### Oral glucose tolerance test (OGTT)

In the 18th week of the experiments, the rats were fasted (12 h) and blood samples were collected from the caudal vein at 0, 30, 60, and 120 min after administration by gavage of a dextrose solution (2 g/kg of weight). Blood glucose was analyzed using the Accu-Chek Active^®^ digital meter (Roche Diagnostics GmbH, Germany). Results are reported as the area under the curve.

### Tissue and blood collection

At the end of the 18 weeks of experiment, the animals were fasted (12 h) and euthanized by inhalation of carbon dioxide (100% of carbon dioxide gas at a gradual fill rate of 20−30% of the chamber volume per minute) followed by laparotomy. Blood samples were collected in a dry tube for biochemical analysis. After blood coagulation, the samples were centrifuged (2044 *g* for 10 min at 4°C) for serum separation and maintained at −80°C for biochemical analysis. Adipose tissue deposits (inguinal, retroperitoneal, epididymal, and brown) and kidneys were dissected and weighed. Right kidney fragments were collected in histological cassettes for further processing and preparation of histological slides.

### Adiposity assessment

The Lee Index (LI) was calculated using body weight and naso-anal length of animals (16), and white adipose fat pads (inguinal, retroperitoneal, epididymal) were weighed for calculations of adiposity (17). The equations for each index are described below:

$$LI=\sqrt[{3}]{\frac{body\;weight\;(g)}{naso-anal\;length\;(cm)}}1000$$ (Eq. 1)

$$Adiposity=\frac{\sum of\;weight\;white\;adipose\;fat\;pads\;(g)}{body\;weight\;(g)}\times 100$$ (Eq. 2)

### Biochemical analysis

Biochemical analyses were performed to evaluate serum concentration of urea, creatinine, total cholesterol, high density lipoprotein (HDL), triglycerides, and glucose. Analyses were performed in technical triplicates by commercial kits of the Bioclin/Quiabasa^®^ Laboratory (Brazil) according to the manufacturer's protocol, using an automated analyzer (Wiener Lab, CM 200, Brazil) at the Laboratory of Clinical Analysis of Pharmacy School of UFOP.

### Kidney lipid extraction and quantification

Kidney lipid extraction was performed according to the protocol adapted from Folch et al. (18). One hundred milligrams of kidney tissue was homogenized with a chloroform/methanol solution (2:1) followed by vigorous stirring. Subsequently, 400 μL of methanol was added to each sample and centrifuged for 10 min at 3370 *g* at 4°C. The supernatant was homogenized with 800 μL of chloroform and 640 μL of sodium chloride solution (0.73%) followed by centrifugation (3370 *g* at 4°C for 10 min). The supernatant was discarded and the tube was washed 3 times with 600 μL of Folch's solution (3% chloroform solution, 48% methanol, 47% distilled water, and 2% NaCl (0.29%)). The supernatant was discarded again and the solvent traces were dried in a semi-open oven at 40°C until complete evaporation. The quantity amount of lipids extracted was calculated using the following equation:

$$Lipids(\%)=\frac{\left(final\;tube\;weight-initial\;tube\;weight\right)}{tissue\;weight}\times 100$$ (Eq. 3)

The dry tube was weighed, and the lipids were resuspended in 500 μL of isopropanol. The lipidic fraction was determined in the renal tissue through the dosages of total cholesterol and triglycerides using commercial kits (Bioclin, Brazil), according to the manufacturer's instructions.

### Histological analysis of kidney

Kidney sections were fixed in methanol-DMSO solution (8:2), processed, and paraffin-embedded. Histological sections (4-μm thick) were stained with Masson's trichrome. Thirty images were visualized with the 40× objective and digitized by the Leica DFC340FX microchamber (Leica Microsystems GmbH, Germany) associated with the Leica DM500 microscope (Leica Microsystems GmbH). The area measurements of the renal corpuscle and glomerulus were obtained with the aid of the FIJI software (ImageJ, USA), using the interactive or freehand measurement tool. The stability test was performed to determine the average number of glomeruli to be analyzed. The urinary space (Bowmnan's space) area was calculated by subtracting the area of the glomerulus from the area of the renal corpuscle. To determine the presence of fibrosis, the area occupied by collagen in all compartments of the kidney (cortex and medulla) was quantified using Leica Qwin software, where RGB color detection algorithms were applied to detect colors by comparing the intensity of each channel in a given pixel to identify and track regions of specific colors in an image. During the quantitative assessment, total collagen was evaluated using as a “normalizer” or “cut-off” of the collagen area values observed in control animals. All histological analyses were performed at Multiuser Laboratory of Microscopy of the Nucleus of Biological Sciences Research of UFOP.

### Gene expression analysis by quantitative reverse transcriptase polymerase chain reaction (RT-qPCR)

Total RNA was extracted from 100 mg of renal tissue using Trizol (Invitrogen, Brazil) and chloroform (Merck, Brazil). The purification was performed by SV Total RNA Isolation System kit (Promega, Brazil). The total RNA concentration was determined by spectrophotometry (NanoDrop Lite, Thermo 226 Scientific, USA), and RNA integrity was analyzed by 1.2% agarose gel electrophoresis. The cDNA was synthesized from 1 μg of total RNA using the High Capacity cDNA Reverse Transcription Kit (Applied Biosystems, USA), according to the manufacturer's instructions. mRNA expression was quantified by qPCR using 3 μL of specific primers (mix forward and reverse at 2.5 μM each), 2 μL of cDNA (50 ng/μL), and 5 μL of GoTaq^®^ qPCR Master Mix (Promega, USA) in an ABI Prism 7300 Sequence Detection System (Applied Biosystems) with the following reaction conditions: 95°C for 2 min, followed by 40 cycles of 95°C for 15 s and 60°C for 1 min, with an additional dissociation stage of 95°C for 15 s, 60°C for 1 min, 95°C for 15 s, and 60°C for 15 s. The relative gene expression was calculated through the 2^−ΔΔCq^ method using hypoxanthine phosphoribosyltransferase 1 (*Hprt1*) as normalizing gene. Primer sequences for rat transcripts were as follows: *Hprt1* (Forward: 5′ GCA GAC TTT GCT TTC CTT GG 3′; Reverse: 5′ ATC CAA CAC TTC GAG AGG TCC 3′), *Sirt1* (Forward: 5′ GGT TGC AGG AAT CCA AAG G 3′; Reverse: 5′ CCA CGA ACA GCT TCA CAA TC 3′), *Sirt2* (Forward: 5′ CAC GAT GAG CTG GAT GAA AG 3′; Reverse: 5′ CGG GCT TTA CCA CAT TCT G 3′), *Sirt3* (Forward: 5′ GGG TCC TTT GCT CTG AGT CC 3′; Reverse: 5′ TCC ACC AGC CTT TCC ACA C 3′), *Sirt4* (Forward: 5′ TTG ATT TCA TCC GCA GTG 3′; Reverse: 5′ CCC AAG TTT CTC CCA GTT 3′), *Sirt5* (Forward: 5′ AAC GCA AAG CAC ATA GTC AT 3′; Reverse: 5′ AGC AAA GGC CAG AGG AGT 3′), *Sirt6* (Forward: 5′ GCC GTC TGG TCA TTG TCA 3′; Reverse: 5′ AGC CTT GGG TGC TAC TGG 3′), and *Sirt7* (Forward: 5′ AGC ACG GCA GCC TCT ATC 3′; Reverse: 5′ AGG TCG GCA GCA CTC ACA 3′).

### Statistical analysis

Statistical analyses were carried out through GraphPad Prism, version 6.01 (GraphPad Software, USA). Data were analyzed by two-way ANOVA, followed by Bonferroni post-test, and the results are reported as means±SD, considering significant values of P<0.05.

## Results

### Regular swimming training with a workload partially inhibited the obesogenic effect induced by prolonged HSD consumption

Supplementary Table S1 presents the results of the biometrics parameters of the experimental groups. Regarding the total caloric intake, our results revealed a lower food intake in animals from the HSD group compared to the STD group (P<0.0001), with regular swimming training with a workload having a potentiating effect on this event (P<0.0001). Despite the lower caloric intake, animals fed HSD for 18 weeks developed a picture of obesity, characterized by an increase in body mass gain (P<0.0001), white adipose tissue mass (epididymal, inguinal, and retroperitoneal adipose tissue) (P<0.0001), and Lee’s (P<0.0032) and adiposity (P<0.0001) indexes compared to the control diet groups fed STD, sedentary and trained (S-STD and T-STD). However, in HSD-fed animals, swimming training with a workload was responsible for a protective effect on the development of obesity, preventing body mass gain (P<0.0001), increased white adipose tissue deposits (P<0.0001), and increased Lee’s (P<0.0032) and adiposity (P<0.0001) indexes, these values being comparable with those of the S-STD groups. Regarding the kidneys, no changes were observed in the mass of this organ among the experimental groups in study.

The data referring to the serum biochemical parameters of the experimental groups are reported in Table 1. These results showed that the consumption of the HSD by sedentary animals for 18 weeks significantly increased serum levels of triglycerides (P<0.0001) and the area under the curve in the OGTT (P=0.002) compared to control groups (S-STD and T-STD). However, regular swimming training with a workload was not able to reverse the deleterious metabolite effects of HSD on triglyceride levels (P=0.682) and on the development of glucose intolerance (observed by the OGTT test) (P=0.200). Furthermore, animals fed the HSD showed a decrease in a serum urea level, with an effect of diet (P=0.003) and physical training (P=0.025). Regarding blood glucose, although no significant changes were observed among the experimental groups, the results pointed to an effect of diet (P=0.009) on this metabolic parameter.

**Table 1:** Serum biochemistry parameter of Wistar rats of the experimental groups after 18 weeks.

Parameters	Groups				P-value		
	S-STD	T-STD	S-HSD	T-HSD	Effect of diet	Effect of training	Interaction
Urea (mg/dL)	72.20±6.47	60.92±6.75	58.58±4.99^a^	56.21±8.49^a^	0.003	0.025	0.130
Creatinine (mg/dL)	0.72±0.11	0.78±0.09	0.76±0.11	0.95±0.27	0.077	0.047	0.255
Cholesterol (mg/dL)	83.92±12.88	77.75±14.76	77.62±14.08	71.91±15.73	0.255	0.265	0.965
HDL (mg/dL)	26.34±2.07	24.43±5.45	23.07±4.13	24.24±4.95	0.262	0.808	0.318
Triglyceride (mg/dL)	73.40±10.69	71.64±17.74	123.62±32.53^a,b^	116.85±36.14^b^	<0.0001	0.682	0.809
Glucose (mg/dL)	115.80±31.38	105.26±15.71	130.72±22.92	134.53±25.67	0.009	0.678	0.374
OGTT/AUC (mg/dL)	15139±977	13815±2661	17693±3248^a,b^	16845±1829^b^	0.002	0.200	0.776

Data are reported as means±SD. Values of P<0.05 were considered significant. ^a^P<0.05 compared to the S-STD group; ^b^P<0.05 compared to the T-STD group (two-way ANOVA followed by the Bonferroni post-test; n=10/group). OGTT/AUG: Oral glucose tolerance test/area under the curve; S-STD: sedentary standard chow diet; T-STD: trained standard chow diet; S-HSD: sedentary high-sugar diet; T-HSD: trained high-sugar diet.

### Effects of HSD and regular swimming training with a workload on lipid deposition in kidneys

**Figure 1:**
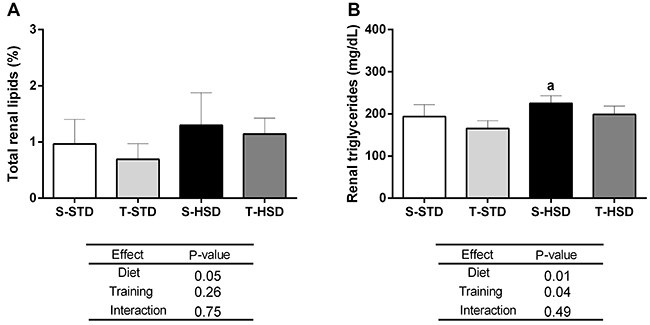
Renal lipid content in Wistar rats from the experimental groups after 18 weeks. **A**, Total percentage of kidney lipid content. **B**, Triglycerides (mg/dL) content in the kidney. Data are reported as means±SD. Values of P<0.05 were considered significant (two-way ANOVA followed by the Bonferroni post-test; n=10/group). ^a^P<0.05 compared to the T-STD group. S-STD: sedentary standard chow diet; T-STD: trained standard chow diet; S-HSD: sedentary high-sugar diet; T-HSD: trained high-sugar diet.

The results regarding the impact of HSD and regular swimming training with a workload on the renal lipid content are shown in Figure 1. Regarding the amount of total lipids evaluated by the Folch methodology, no statistical difference was detected among the experimental groups, although our results showed increased levels of renal triglycerides in the S-HSD group compared to the T-STD group. More specifically, our results indicated an effect of diet (P=0.01) and physical training (P=0.04) on renal triglyceride levels.

### Impact of HSD and regular swimming training with a workload on renal function

Table 2 presents the results of biochemical analyses of urine metabolites. No changes were observed in urinary proteinuria levels and in the proteinuria/creatinuria ratio in the experimental groups under study. There were also no significant differences in relation to CrCl among the groups.

**Table 2:** Urinary biochemical parameters of Wistar rats of the experimental groups after 18 weeks.

Parameters	Groups				P-value		
	S-STD	T-STD	S-HSD	T-HSD	Effect of diet	Effect of training	Interaction
Proteinuria (mg/dL)	78.36±18.45	75.73±12.15	108.54±32.81	129.13±52.96	0.585	0.020	0.48
Proteinuria/creatinuria	0.81±0.28	0.68±0.41	0.91±0.71	0.64±0.39	0.51	0.19	0.16
CrClr	0.240±48.8	0.267±82.24	0.312±71.7	0.364±97.3	0.30	0.24	0.67

Data are reported as means±SD. Values of P<0.05 were considered significant (two-way ANOVA followed by the Bonferroni post-test; n=10/group). S-STD: sedentary standard chow diet; T-STD: trained standard chow diet; S-HSD: sedentary high-sugar diet; T-HSD: trained high-sugar diet; CrCl: creatinine clearance (in mL/min corrected by body mass index per 100 g of body weight).

**Figure 2:**
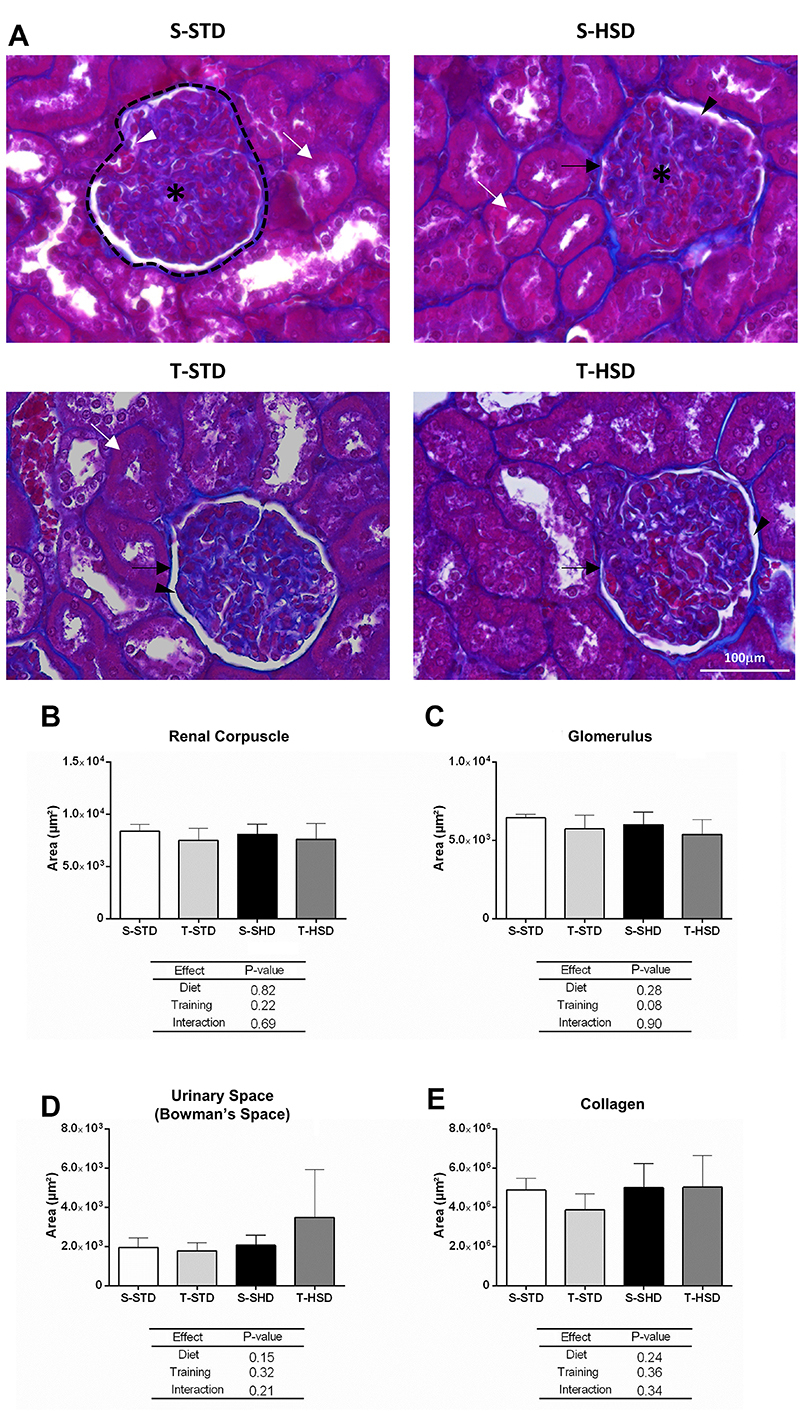
Histological analyses of renal tissues of Wistar rats submitted to the experiment for 18 weeks. **A**, Photomicrographs of renal tissue histological sections at 200× magnification, Masson's trichrome staining. Dashed line: renal corpuscle (urinary space + glomerulus); asterisks: glomerulus; black arrow: Bowman's capsule; black arrowhead: urinary space; white arrowhead: afferent and efferent arterioles; white arrow: renal tubules; collagen is stained blue. Scale bar=100 µm. **B**, Renal corpuscle area (µm^2^). **C**, Glomerulus area (µm^2^). **D**, Urinary space (µm^2^). **E**, Collagen area (µm^2^). Data are reported as means±SD. Values of P<0.05 were considered significant (two-way ANOVA followed by the Bonferroni post-test; n=10/group). S-STD: sedentary standard chow diet; T-STD: trained standard chow diet; S-HSD: sedentary high-sugar diet; T-HSD: trained high-sugar diet.

Possible renal alterations induced by HSD and the role of swimming training in this event were evaluated at the tissue level in this study through histopathological analysis of the kidney. The images in Figure 2A represent the histology of the kidney of the experimental groups. As can be seen, no tissue damage and/or renal fibrosis (Figure 2A) was identified among groups up to 18 weeks of experimental intervention. Furthermore, the quantitative analysis of photomicrographs of kidney sections also did not reveal statistical differences in relation to renal corpuscle area (Figure 2B), glomerular area (Figure 2C), urinary space (Figure 2D), and collagen area (Figure 2E).

### HSD and regular swimming training with a workload changed the sirtuin expression profile in renal tissue

**Figure 3:**
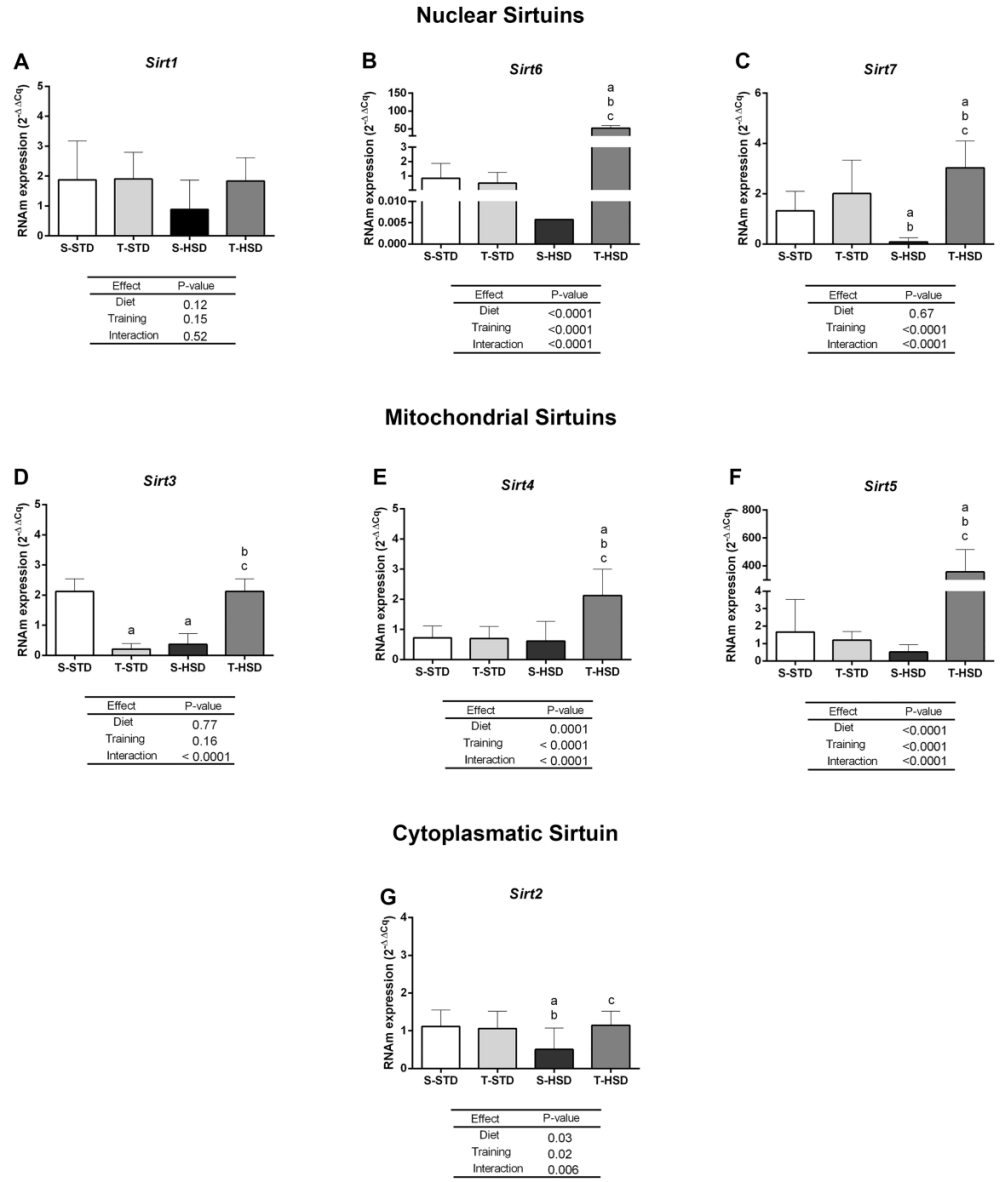
Effect of a high-sugar diet (HSD) and swimming training with workload over an 18-week period on expression profile of *Sirts* (*Sirt1-7*; **A**-**G**) in the renal tissue of Wistar rats. Data are reported as means±SD. ^a^P<0.05 compared to the S-STD group, ^b^P<0.05 compared to the T-STD group, and ^c^P<0.05 compared to the S-HSD group (two-way ANOVA followed by the Bonferroni post-test; n=10/group). S-STD: sedentary standard chow diet; T-STD: trained standard chow diet; S-HSD: sedentary high-sugar diet; T-HSD: trained high-sugar diet.

Figure 3 shows gene expression of *Sirts1-7* in the kidney of rats submitted to HSD or STD with and without regular swimming training during an 18-week period. No significant changes were identified in the expression of *Sirt1*, with the expression of this gene being constant among experimental groups (Figure 3A). The consumption of HSD in sedentary animals (S-HSD group) led to downregulation of *Sirt2*, however physical training reversed this effect, restoring the expression of *Sirt2* to levels compatible with animals fed the STD diet (Figure 3G); there was an effect of diet (P=0.03), physical training (P=0.02), and the interaction between these variables (P=0.006).

Regarding *Sirt3*, the results indicated a decrease in the expression of this gene in trained animals fed STD (T-STD group) and sedentary animals fed HSD (S-HSD group). However, in animals fed HSD, training (T-HSD group) induced an upregulation of *Sirt3* to levels compatible with animals in the S-STD group. These results were associated with a significant effect of the interaction between diet and swimming training with a workload (P<0.0001) (Figure 3D).

The association between HSD and regular swimming training with a workload (P<0.0001) in the T-HSD group induced an expressive positive modulation on the expressions of *Sirt4*, *Sirt5*, and *Sirt6*, in relation to the other groups, which showed stable expressions of these genes (Figure 3E, F, and B, respectively). Regarding *Sirt7*, the consumption of HSD by sedentary animals (S-HSD group) led to a decrease in the expression of this gene, but HSD and swimming training with a workload (P<0.0001) induced an increase in the expression of *Sirt7* in relation to the other experimental groups (Figure 3C).

## Discussion

In the present study, we first performed the characterization of the experimental model through the evaluation of the behavioral profile of food consumption and the analysis of the morphometric and biochemical parameters of the experimental animals. Our results revealed that prolonged consumption of HSD, even at a lower total caloric intake, led to the development of obesity (as evaluated by the increase in body mass, white adipose tissue deposits, and Lee's and adiposity indices) and metabolic changes (hypertriglyceridemia and glucose intolerance). As previously published by our research group, post-weaning exposure of Wistar rats to HSD in isocaloric condition can promote fat mass/body mass increase and metabolic syndrome phenotype in adulthood (13-15,19). In fact, studies have shown that the consumption of diets high in sugar can lead to the development of obesity regardless of higher energy consumption (13,20). As discussed by de Oliveira et al. (13), the increase in fat mass gain together with a lower caloric consumption of HSD may be associated with the deleterious effects of sugar on the physiological, neurohormonal, metabolic, and biochemical pathways. In addition, we highlight that the lower serum urea concentration found in animals that consumed HSD was probably related to the lower protein content of this diet compared to STD, since urea is a metabolite of dietary protein. The complete nutritional composition of experimental diets was previously published. Furthermore, the lower concentration of urea in these animals may be associated with protein malnutrition caused by HSD. We also showed that workload swimming training partially inhibited the HSD-induced obesogenic effect, leading to a reduction in HSD-induced fat mass gain.

In a second stage of the present study, we evaluated the functionality and tissue integrity of the kidneys. Although the consumption of HSD for the experimental period of 18 weeks was efficient in inducing obesity, kidney damage was not identified. In fact, the association between obesity and development and progression of CKD is complex, as both conditions share pathophysiological pathways, risk factors, and associated diseases (21). The exact mechanisms underlying this association are still unclear (22). Approximately 10-25% of obese individuals present metabolic changes and only a small proportion of obese individuals develop CKD, which suggests that the increase in fat mass alone is not enough to induce kidney damage (22,23). More specifically, in the present study, no differences were identified in terms of kidney mass or total lipid content in the kidney. However, our results showed an increase in renal triglycerides in the S-HSD group compared to the T-STD group, demonstrating a protective effect of physical exercise on the accumulation of triglycerides in the kidneys of animals fed HSD. This is of clinical importance since the accumulation of triglycerides in the kidneys can lead to lipotoxicity, inflammation, oxidative stress, and mitochondrial dysfunction and lead to significant deleterious effects on kidney function in the long term (24). In addition, no changes were identified in histological analyses, in urinary metabolism, and in the estimation of glomerular filtration rate (measured here by CrCl). Taken together, our results suggested that kidney function appeared to be preserved in the experimental groups. However, a trend towards an increase in urinary metabolites (proteinuria and proteinuria/creatinuria ratio) was observed in the HSD groups, which can indicate the incipient loss of renal homeostasis.

In a third stage of this study, we evaluated the impact of HSD consumption and regular swimming training with a workload on transcriptional modulation of renal *Sirts*. Our results revealed a distinct expression profile in relation to the nuclear *Sirts* (*Sirt1*, *Sirt6*, and *Sirt7*). Nuclear SIRTs can act in chromatin remodeling by catalyzing post-translational modification of histone and non-histone proteins, including transcription factors, thus playing an important role in the control of gene expression (25). In the present study, *Sirt1* was the only gene that showed no change in its expression between the experimental groups. In the kidneys, Sirt1 exerts a protective action against tubular cell damage and kidney injury, in addition to delaying fibrogenesis and renal aging (26). Thus, in the present study, we raised the hypothesis of a possible association between the absence of fibrosis or tissue damage in the kidneys and the unaltered expression of *Sirt1* during the 18 weeks of HSD consumption. Regular physical exercise was also unable to modulate *Sirt1* expression during this experimental period. On the other hand, our results revealed that the interaction between HSD and training had a potentiating effect, inducing a significant increase in *Sirt6* and *Sirt7* expression in the kidneys. Studies show that overexpression of *Sirt6* and *Sirt7* attenuates kidney injury by regulating inflammation and apoptosis (27-29). Liu et al. (30) also showed that downregulation of *Sirt6* was related to an increase in proteinuria. Here, we speculated that the increase of *Sirt6 and Sirt7* expression in trained animals fed HSD may represent a beneficial adaptive response (protective effect) to HSD consumption in the long term.

SIRT2 is the main cytosolic SIRT, but it can also be found in the nucleus and mitochondria (3). In contrast to the other SIRTs that have a renoprotective effect, *Sirt2* has been shown to promote kidney injury, induced by chemical compounds, and diabetic nephropathy by decreasing cellular autophagy and increasing apoptosis and inflammation. However, it has been reported that *Sirt2* expression decreased in the hyperglycemic state (7,31). Thus, the precise biological mechanism related to SIRT2 in kidney damage is still unclear. Our results revealed that HSD led to a downregulation of renal *Sirt2* expression. However, training restored the expression levels of *Sirt2* to similar values found in animals fed STD.

*Sirt3* is the main promoter of mitochondrial energy metabolism and has the most robust deacetylase activity of all mitochondrial *Sirts* (3). In the kidneys, *Sirt3* is thought to be a master regulator of protection against acute kidney injury, and its increase is important for improving mitochondrial dynamics. Furthermore, both oxidative stress and mitochondrial damage lead to acute kidney injury and are associated with reduced levels of renal *Sirt3* (32). Here we found that renal *Sirt3* expression was downregulated by HSD intake in sedentary animals, suggesting that this low expression of *Sirt3* may be an initial response to loss of renal homeostasis, although renal damage is not yet present in these animals. In fact, deregulation of SIRTs induced by overfeeding and/or unhealthy diets can lead to changes in metabolism, compromising antioxidant defense and consequently favoring the development of metabolic diseases (3). Our results also revealed that HSD combined with physical exercise restored *Sirt3* expression to a similar level as in the control group, suggesting that exercise may play an important role in preventing and delaying potential damage caused by HSD in the long term. Surprisingly, however, *Sirt3* expression was decreased by training in the control group fed STD. It is known that physical exercise improves renal metabolism (33), but little is yet known about the effect of physical training on *Sirt3* renal expression. It has been demonstrated that exercise has a positive effect on Sirts expression, including *Sirt3*, on cardiac (34) and skeletal muscle (35). However, Palacios et al. (36) showed that *Sirt3* expression is induced by exercise in a tissue-specific manner since training increased *Sirt3* expression in skeletal muscle but not in cardiac muscles.

In the present study, the mitondrial *Sirt4* and *Sirt5* showed similar expression profiles. Both *Sirts* were upregulated in the kidney by the interaction between HSD and physical training. In this context, overexpression of *Sirt4* protects against diabetic nephropathy by inhibiting apoptosis in a glucose-induced mouse podocyte model (37). Regarding SIRT5, the general importance of this enzyme is still poorly understood and even less is known about its roles in renal homeostasis. However, SIRT5 seems to be involved in many aspects of mitochondrial function, including the urea cycle, energy metabolism, and prevention of damage from oxidative stress (3). Like *Sirt6*, the expression of *Sirt4* and *Sirt5* remained constant in sedentary animals fed HSD compared to the control group fed STD. In this sense, we speculate that the consumption of HSD for 18 weeks was not able to have a modulating effect on the expression of these *Sirts*. However, the increase in the expression of these *Sirts* in animals subjected to physical exercise may represent a positive long-term response to possible injuries triggered by the consumption of HSD in the long term. However. future studies are needed to elucidate this hypothesis.

**Figure 4:**
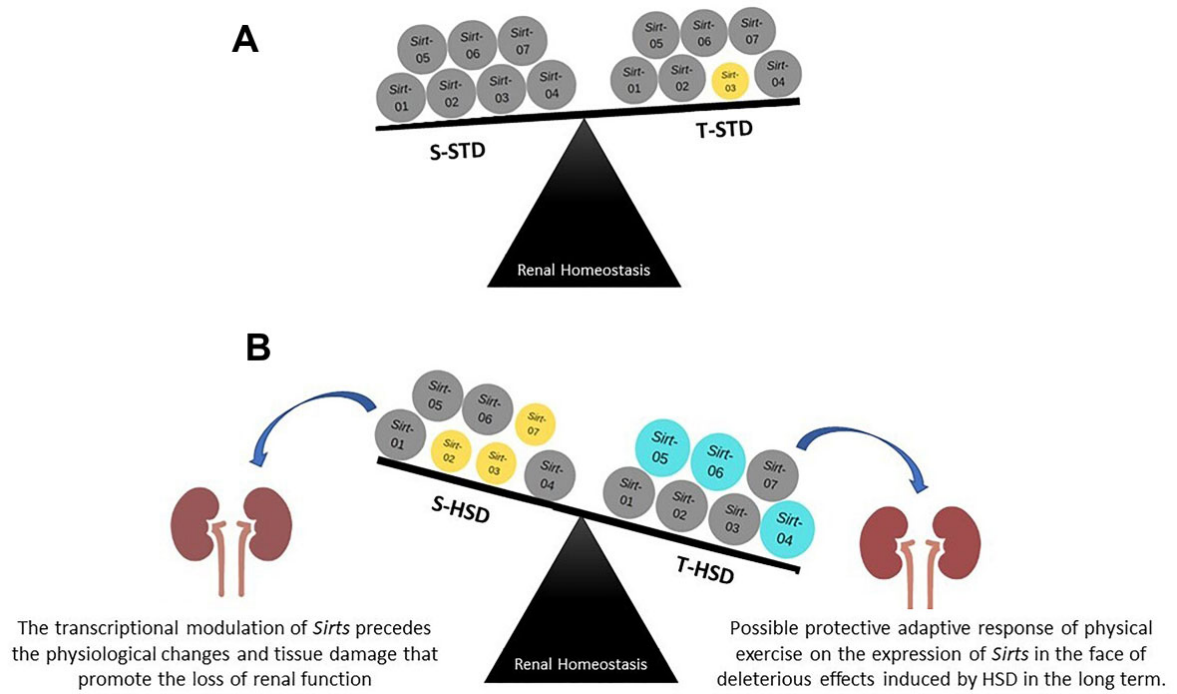
Overview of the modulation of kidney sirtuins expression by high-sugar diet (HSD) and regular swimming. **A**, Overview of the modulation of renal sirtuin expression in STD-fed rats. **B**, Overview of the modulation of renal sirtuin expression in HSD-fed rats. The expression of sirtuins (*Sirt*1-7) in each experimental group are shown as circles. Yellow circles represent low expression and blue circles represent high expression in relation to gray circles. S-STD: sedentary standard chow diet; T-STD: trained standard chow diet; S-HSD: sedentary high-sugar diet; T-HSD: trained high-sugar diet.

An overview of the expression profile of renal *Sirts* in the different experimental groups as well as their physiological relationship observed in the kidney is presented in Figure 4.

In conclusion, our results revealed that, with the exception of *Sirt1*, all the other renal *Sirts* were transcriptionally modulated by HSD, training, and/or the interaction of these two variables. These results indicated that molecular events represented by *Sirts* modulation may precede the physiological changes and tissue damage that led to the loss of kidney function. The expression of renal *Sirts* during the development and progression of renal damage induced by high sugar consumption might be a dynamic process, as is the protective adaptive response of exercise to expression given the potential deleterious effects induced by HSD in the long term.

## Supplementary Material

Click to view [pdf].
