# Supplementary material for: Modulation of sirtuin expression by a high-sugar diet and regular swimming trained precedes the loss of kidney function
Source: Braz J Med Biol Res. 2025 Jan 31;58:e13043. doi: 10.1590/1414-431X2024e13043 (PMC11793145; doi:10.1590/1414-431X2024e13043)
Supplement: Supplementary file 1 [file 1414-431X-bjmbr-58-e13043-suppl.pdf]

Supplementary Table S1. Food consumption and morphometric parameters of Wistar rats of the experimental groups after 18 weeks.

Parameters	Groups				P-value		
	S-STD	T-STD	S-SHD	T-SHD	Effect of diet	Effect of training	Interaction
Total food consumption (Kcal)	4229.43 ± 12.81	4324.95 ± 12.01[a]	4052.63 ± 14.15[a,b]	3814.61 ± 12.13[a,b,c]	<0.0001	<0.0001	<0.0001
Initial weight (g)	58.42 ± 3.91	62.00 ± 5.20	59.55 ± 4.97	60.50 ± 5.08	–	–	–
Final weight (g)	398.57 ± 23.99	375.50 ± 30.50	496.88 ± 29.98[a,b]	430.30 ± 46.73[b,c]	<0.0001	0.0005	0.0713
Body weight gain (g)	320.86 ± 34.64	293.70 ± 39.28	422.33 ± 42.91[a,b]	373.80 ± 43.18[b,c]	<0.0001	0.0051	0.4056
Epididymal AT (g)	5.492 ± 1.30	4.759 ± 1.257	14.99 ± 3.42[a,b]	7.82 ± 3.53[c]	<0.0001	<0.0001	0.0003
Inguinal AT (g)	9.28 ± 2.25	7.17 ± 1.65	20.93 ± 3.63[a,b]	11.69 ± 5.09[b,c]	<0.0001	<0.0001	0.0022
Retroperitoneal AT (g)	5.18 ± 1.41	4.15 ± 1.46	16.56 ± 3.43[a,b]	8.80 ± 3.70[c]	<0.0001	<0.0001	0.0016
Lee Index	0.31 ± 0.01	0.31 ± 0.01	0.33 ± 0.01[a,b]	0.31 ± 0.01[c]	0.0032	0.0032	0.0032
Adiposity Index	4.98 ± 1.04	4.27 ± 0.55	10.52 ± 1.47[a,b]	6.62 ± 2.14[b,c]	<0.0001	<0.0001	0.0011
Right kidney (g)	1.28 ± 0.18	1.22 ± 0.07	1.32 ± 0.10	1.23 ± 0.10	0.4788	0.0638	0.7557
Left kidney (g)	1.28 ± 0.20	1.21 ± 0.08	1.34 ± 0.11	1.21 ± 0.12	0.5011	0.0683	0.5690

Data are reported as means±SD. [a]P<0.05 compared to the S-STD group, [b]P<0.05 compared to the T-STD group, and [c]P<0.05 compared to the S-HSD group (two-way ANOVA followed by the Bonferroni post-test; n=10/group) S-STD: sedentary standard chow diet; T-STD: trained standard chow diet; S-HSD: sedentary high-sugar diet; T-HSD: trained high-sugar diet; AT: adipose tissue.
